# TFE: A Transformer Architecture for Occlusion Aware Facial Expression Recognition

**DOI:** 10.3389/fnbot.2021.763100

**Published:** 2021-10-25

**Authors:** Jixun Gao, Yuanyuan Zhao

**Affiliations:** ^1^Department of Computer Science, Henan University of Engineering, Zhengzhou, China; ^2^Department of Computer Science, Zhengzhou University of Technology, Zhengzhou, China

**Keywords:** affective computing, facial expression recognition, occlusion, transformer, deep learning

## Abstract

Facial expression recognition (FER) in uncontrolled environment is challenging due to various un-constrained conditions. Although existing deep learning-based FER approaches have been quite promising in recognizing frontal faces, they still struggle to accurately identify the facial expressions on the faces that are partly occluded in unconstrained scenarios. To mitigate this issue, we propose a transformer-based FER method (TFE) that is capable of adaptatively focusing on the most important and unoccluded facial regions. TFE is based on the multi-head self-attention mechanism that can flexibly attend to a sequence of image patches to encode the critical cues for FER. Compared with traditional transformer, the novelty of TFE is two-fold: (i) To effectively select the discriminative facial regions, we integrate all the attention weights in various transformer layers into an attention map to guide the network to perceive the important facial regions. (ii) Given an input occluded facial image, we use a decoder to reconstruct the corresponding non-occluded face. Thus, TFE is capable of inferring the occluded regions to better recognize the facial expressions. We evaluate the proposed TFE on the two prevalent in-the-wild facial expression datasets (AffectNet and RAF-DB) and the their modifications with artificial occlusions. Experimental results show that TFE improves the recognition accuracy on both the non-occluded faces and occluded faces. Compared with other state-of-the-art FE methods, TFE obtains consistent improvements. Visualization results show TFE is capable of automatically focusing on the discriminative and non-occluded facial regions for robust FER.

## 1. Introduction

Facial expressions are the most natural way for humans to express emotions. Facial expression recognition (FER) has received significant interest from psychologists and computer scientists as it facilitates a number of practical applications, such as human-computer interaction, pain estimation, and affect analysis. Although current FER systems have obtained promising accuracy when recognizing facial images captured in controlled scenarios, these FER systems usually suffer from considerable performance degradation when recognizing expressions in the wild conditions. To fill the gap between the FER accuracy on the controlled faces and in-the-wild faces, researchers start to collect large-scale facial expression databases in uncontrolled environment (Li et al., 2017; Mollahosseini et al., 2017). Despite the usage of face images in the uncontrolled scenario, FER is still challenging due to the existence of facial occlusions. It is non-trivial to solve the occlusion problem because facial occlusions are various and abundant. These facial occlusions may appear in many forms, such as breathing masks, hands, drinks, fruits, and other objects that might appear in front of the human faces in our daily life. The facial occlusions may block any other part of the face, and the variability of occlusions would inevitably induce the decreased FER performance.

Previous studies usually handled FER under occlusion with sub-region-based features (Kotsia et al., 2008; Li et al., 2018a,b; Wang et al., 2020b), e.g., Kotsia et al. (2008) presented a detailed analysis on occluded FER and conclude that FER will suffer from more decreased performance with occluded mouth than the occluded eyes. With the popularity of the data-driven convolutional neural network (CNN) techniques, a number of recent efforts on FER have been made on the collection of large-scale facial expression databases and exploit CNN to enhance the performance of FER. Li et al. (2018a) proposed to decompose facial regions in the convolutional feature maps with the manually defined facial landmarks and fused the local and global facial representations *via* attention mechanism. However, the recent CNN-based FER methods lack the ability to learn global interactions and relations between distant facial parts. These methods are not capable of flexibly attending to distinctive facial regions for precise FER under occlusions.

Inspired by the observation (Naseer et al., 2021) that transformers are robust to occlusions, perturbations, and domain shifts, we propose a **T**ransformer Architecture for **F**acial **E**xpression Recognition (TFE) under occlusions. Currently, vision transformers (Dosovitskiy et al., 2020; Li et al., 2021) have demonstrated impressive performance across numerous machine vision tasks. These models are based on multi-head self-attention mechanisms that can flexibly attend to a sequence of image patches to encode contextual cues. The self-attention in the transformers has been shown to effectively learn global interactions and relations between distant object parts. A number of following studies on downstream tasks such as object detection (Carion et al., 2020), segmentation (Jin et al., 2021), and video processing (Girdhar et al., 2019; Fang et al., 2020) have verified the feasibility of the transformers. Given the content-dependent long-range interaction modeling capabilities, transformers can flexibly adjust their receptive field to cope with occlusions in data and enhance the discriminability of the representations.

**Figure 1:**
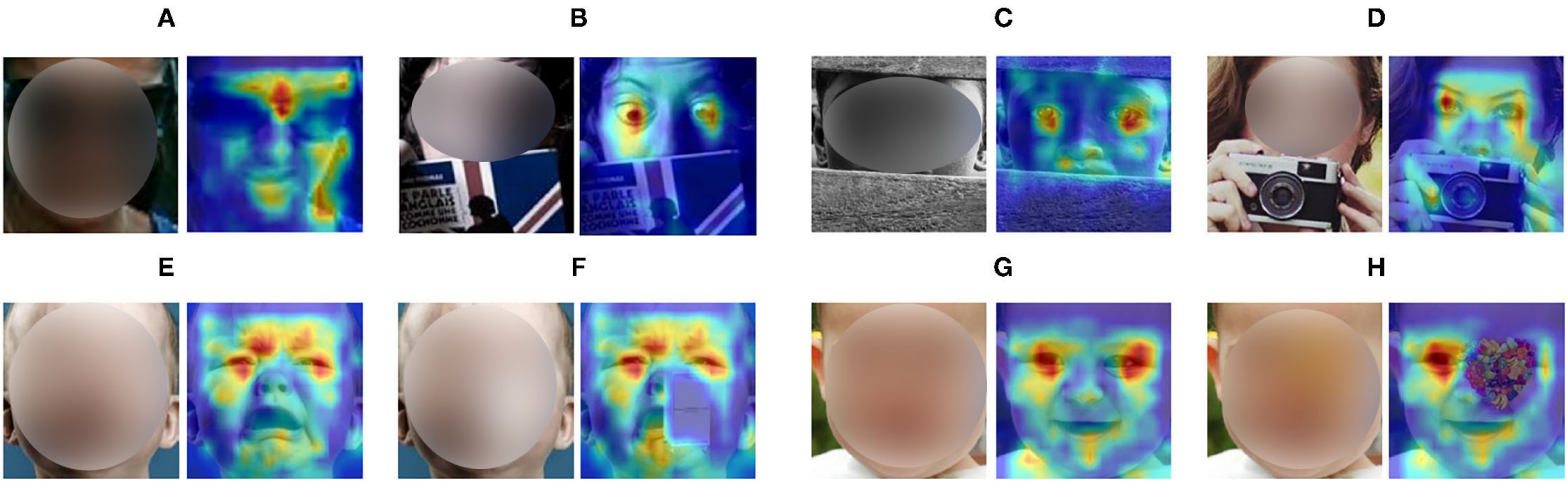
Attention maps of several facial images with real (**A–D** in top row) or synthesized (**E–H** in bottom row) occlusions. Our proposed TFE is capable of perceiving the important facial regions for robust FER. A deep red means high attention. Better viewed in color and zoom in.

Intuitively, human perceives the facial expressions *via* several critical facial regions, e.g., eyes, eyebrows, and corners of the mouth. If some facial patches are occluded, human may judge the expression according to the other highly informative regions. To mimic the way that human recognizes the facial expression, we propose a region selection unit (RS-Unit) that is capable of focusing on the important facial regions. To be specific, RS-Unit selects the discriminative facial regions and removes the redundant or occluded facial parts. We then combine the global classification token with the selected part tokens as the facial expression representation. With the proposed RS-Unit, TFE is able to adaptively perceive the distinctive and unobstructed regions in facial images. To further enhance the discriminability of the representation, we exploit an auxiliary decoder to reconstruct the corresponding non-occluded face. Thus, TFE is capable of inferring the occluded facial regions *via* the unoccluded parts to better recognize the facial expressions. Figure 1 illustrates the attention map of TFE on some facial images. It is clear that TFE is capable of focusing on the critical and unoccluded facial parts for robust FER. More visual examples and explanations can be seen in section 4.2.1.

The contributions of this study can be summarized as follows:

1. We propose a transformer architecture to recognize facial expressions (TFE) from partially occluded faces. TFE consists of a region selection unit (RS-Unit) that automatically perceives and selects the critical facial regions for robust FER. TFE is deployed to focus on the most important and unoccluded facial regions.
2. To further enhance the discriminability of the facial expression representation, TFE contains an auxiliary image decoder to reconstruct the corresponding non-occluded face. The image decoder is merely exploited during the training process and incorporates no extra computation burden at inference time.
3. Qualitative experimental results show the benefits and the advantages of the proposed TFE over other state-of-the-art approaches on two prevalent in-the-wild facial expression databases. Visualization results additionally show that TFE is superior in perceiving the informative facial regions.

## 2. Related Work

We discuss the previous literatures that are related to our proposed TFE, i.e., FER with occlusions and the vision transformer.

### 2.1. Methods for FEE Under Occlusion

For FER tasks, occlusion is one of the inevitable challenges in real-world scenarios. We just classify previous FER methods into two classes: handcrafted features-based methods and deep learning-based approaches.

Early FER under occlusion methods typically encode handcrafted features from face samples, and then learn classifiers based on the encoded features (Rudovic et al., 2012; Zhang et al., 2014). Liu et al. (2013) proposed a novel FER method to mitigate the partial occlusion issue *via* fusing Gabor multi-orientation representations and local Gabor binary pattern histogram sequence. Cotter (2010) introduced to use sparse representation for FER. Especially, Kotsia et al. (2008) analyzed how partial occlusions affect FER performance and found that FER suffers more from mouth occlusion than the equivalent eyes occlusion.

Over the recent years, Convolution Neural Network (CNN) has shown exemplary performance on many computer vision tasks (Schroff et al., 2015; Krizhevsky et al., 2017; Li et al., 2020). The promising learning ability of deep CNN can be attributed to the use of hierarchical feature extraction stages that can adaptively learn the features from the data in an end-to-end fashion. There are many CNN-based FER works (Levi and Hassner, 2015; Ding et al., 2017; Meng et al., 2017; Zeng et al., 2018; Zhang et al., 2018; Li et al., 2019; Jiang et al., 2020). For FER under occlusion, Li et al. (2018a) proposed a CNN with attention mechanism (ACNN) to perceive facial expressions from unoccluded or partially occluded faces. ACNN crops facial patches from the area of important facial features, e.g., mouth, eyes, nose, and so on. The selected multiple facial patches are encoded as a weighed representation *via* a PG-Unit. The PG-Unit calculates the weight of each facial patch according to its obstructed-ness *via* an attention net. Based on this work, Wang et al. (2020b) proposed to randomly crop relative large facial patches instead of small fixed facial parts and refine the attention weights by a region bias loss function and relation-attention module. Ding et al. (2020) proposed an occlusion-adaptive deep network with a landmark-assisted attention branch network to perceive and drop the corrupted local features. Pan et al. (2019) introduced to train two CNNs from non-occluded facial images and occluded faces, respectively. Subsequently, they constrain the distribution of the encoded facial representations from two CNNs to be close *via* adversarial learning.

Our proposed TFE differs from previous CNN-based methods in two ways. One, TFE does not rely on facial landmarks for regional feature extraction. It is because the facial landmarks may show considerable misalignments under severe occlusions. Under this condition, the encoded facial parts are not part-aligned or semantic meaningful. Two, TFE is a transformer-based and the self-attention mechanism in the transformer that can flexibly attend to a sequence of image patches to encode the contextual cues. TFE consists of a region selection unit (RS-Unit) that automatically perceives and selects the critical facial regions for robust FER. TFE is potentially to obtain higher FER accuracy on both non-occluded and occluded faces. We will verify this in section 4.

### 2.2. Vision Transformer

Transformer models have largely facilitated research in machine translation and natural language processing (NLP) (Waswani et al., 2017). Transformer models have become the outstanding standard for NLP tasks. The main idea of the original transformer is to calculate the self-attention by comparing a representation to all other representations in the input sequence. In detail, features are first encoded to obtain memory [including value (*V*) and key (*K*)] and query (*Q*) embedding by linear projections. The product of the query *Q* with keys *K* is used as the attention weights for value *V*. A position embedding is also exploited and added to these representations to introduce the positional information in such a non-convolutional paradigm. Transformers are especially good at modeling long-range dependencies between elements of a sequence.

Inspired by the success of the transformer models, many recent studies try to use transformers in computer vision applications (Dosovitskiy et al., 2020; Li et al., 2021). Among them, Dosovitskiy et al. (2020) applied a pure transformer encoder for image classification. To obtain the input token representations, they crop the input image into 16 × 16 small patches and linearly map the patches to the input dimension of the encoder. Since then, ViTs are gaining rapid interest in various computer vision tasks because they offer a self-attention-based noval mechanism that can effectively capture long-range dependencies. Touvron et al. (2021) showed that ViT models can achieve competitive accuracy on ImageNet with stronger data augmentation and more regularization. Subsequently, transformer models are applied to other popular tasks such as object detection (Carion et al., 2020), segmentation (Jin et al., 2021), and video processing (Girdhar et al., 2019; Fang et al., 2020). In this study, we extend ViT to FER under occlusion and show its effectiveness.

## 3. Method

**Figure 2:**
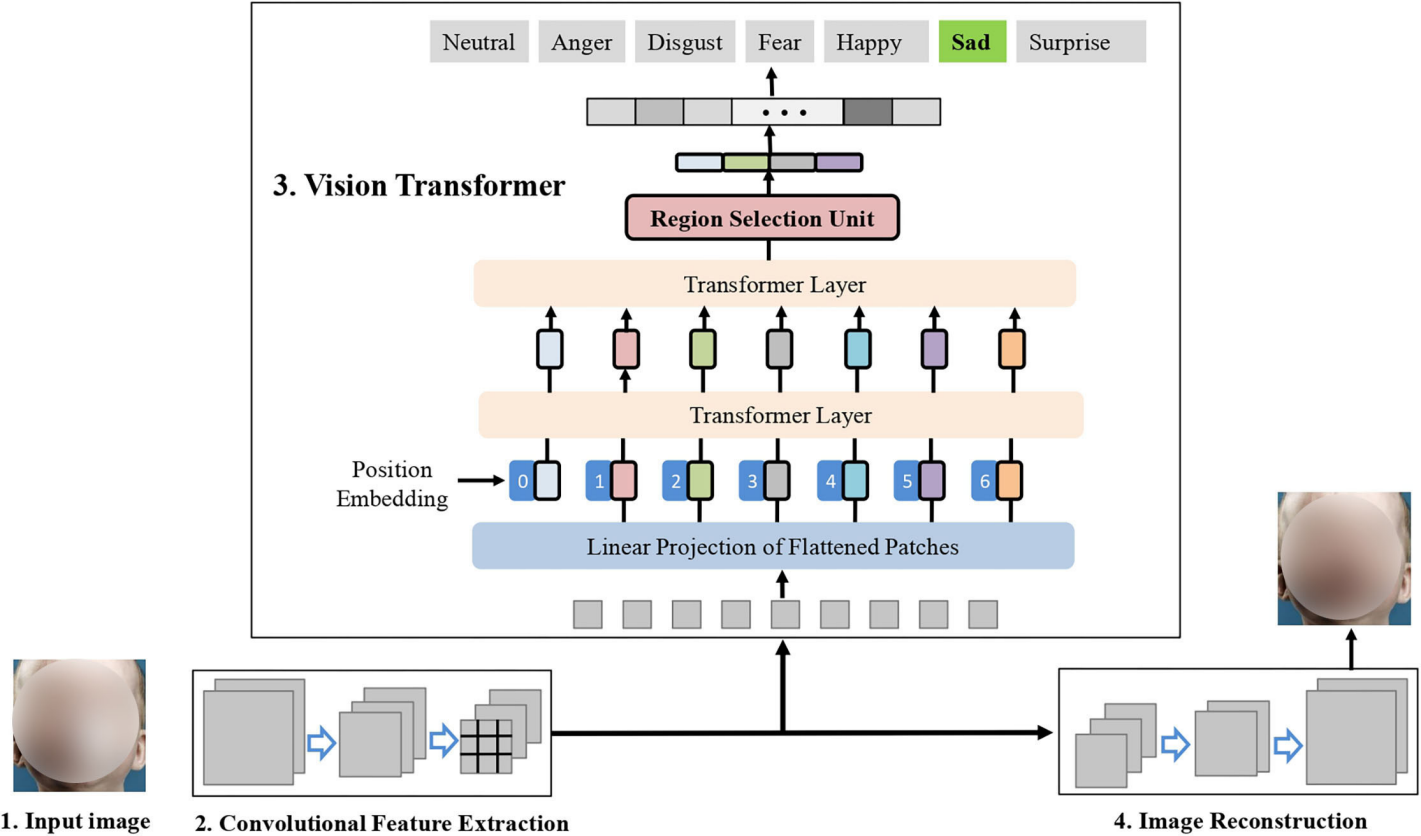
Main idea of the proposed **T**ransformer Architecture for **f**acial **e**xpression recognition (TFE). TFE perceives the informative facial expression representation *via* the vision transformer and the proposed RS-Unit. In the right part, TFE uses an auxiliary decoder to reconstruct the unoccluded faces.

Figure 2 illustrates the main idea of the proposed TFE. Given an input face image, TFE encodes its convolutional feature maps *via* a commonly used backbone network such as ResNet-18 (He et al., 2016). Then, TFE encodes the robust facial expression representation *via* the vision transformer and the proposed RS-Unit. During the training stage, the encoded convolutional feature maps are decoded to reconstruct the unoccluded facial image. Below, we present the details of each of them.

### 3.1. Network Architecture

Following ViT (Dosovitskiy et al., 2020), we first preprocess the input image into a sequence of flattened image patches. However, the conventional split approach merely cuts the images into overlapping or non-overlapping patches, which harms the local neighboring structures and shows substandard optimizability (Xiao et al., 2021). Inspired by Xiao et al. (2021) that exploits a few number of stacked 3×3 convolutions for image sequentialization, we adopt the popular ResNet-based backbone (He et al., 2016) to encode the input facial image **I**. A typical ResNet usually has four stages (Li et al., 2021), and we use the output of the *S*-th stage as the encoded feature maps **X** ∈ ℝ^*H*×*W*×*C*^ feature maps; thus, we get a total of *N* = *H* × *W* image tokens, each token $X_{i}$ with a feature dimension of *C*. As *H* equals *W*, here we use *P* = *H* = *W* for brevity. In our proposed TFE, the image tokens have the spatial size 1 × 1, the input sequence is obtained by: (i) flattening the spatial dimensions of the feature map and (ii) projecting the flattend tokens to the target transformer dimension.

We map the flattend image token **X**_*i*_ into a latent *D*-dimensional feature space *via* a learnable fully connected neural layer. With the sliced image token $X_{i}\in {\mathbb{R}}^{P^{2}\times D},1\leq i\leq N$, a trainable position embedding is plused to the token embeddings to retain positional information as follows:


(1) $$\begin{array}{l} Z_{0}=\left[X_{class};X_{1}\text{E};X_{2}\text{E};X_{N}\text{E}\right]+{\text{E}}_{pos}, \end{array}$$

(2) $$Z_{l}\prime =MSA(LN(Z_{l-1}))+Z_{l-1},\;l\in 1,2,\cdots ,L$$

(3) $$Z_{l}=MLP(LN(Z_{l}\prime ))+Z_{l}\prime ,\;l\in 1,2,\cdots ,L,$$

where *N* means the number of the image tokens, **E** is the token embedding projection, and **E**_*pos*_ means the position embedding. *L* means the number of layers of the multi-head self-attention (MSA) and the multi-layer perceptron (MLP) blocks. The transformer encoder includes alternating layers of multi-head self-attention (MSA) and multilayer perceptron (MLP) blocks. We also add a layernorm (LN) layer before every block and residual connections after every block. Besides, the MLP consists of two fully connected neural layers with a GELU non-linearity. **X**_*class*_ is a classification token that consists of an embedding attached to the sequence of embedded patches. After *L* transformer layers, a classification head is attached to $Z_{L}^{0}$. We implemented the classification with a MLP that consists of one hidden layer at the training and testing phase.

### 3.2. Vision Transformer With RS-Unit

One of the most important problems in FER under occlusion is to precisely perceive the discriminative facial regions that represent subtle facial deformations caused by facial expressions. To this end, we proposed a RS-Unit to automatically select the critical facial parts for robust FER under occlusions. Different with previous methods that use facial landmarks for facial region decomposition (Li et al., 2018a; Ding et al., 2020; Wang et al., 2020b), RS-Unit does not need auxiliary annotation and merely adopts the pre-computed multi-head attention information.

Suppose the model consists of *M* self-attention heads and the hidden features, outputs of the last transformer layer are denoted as $Z_{L}=\left[Z_{L}^{0},Z_{L}^{1},Z_{L}^{2},\cdots \;,Z_{L}^{N}\right]$. To better utilize the attention information, the input to the final classification layer is changed. In detail, the raw attention weights are obtained *via* recursive matrix multiplication in all the layers:


(4) $$\begin{array}{l} a_{total}=\overset{L}{\underset{l=0}{\sum }}a_{l}. \end{array}$$

As **a**_*total*_ spots how information propagates from the preceding transformer layer to the features in the later transformer layers, **a**_*total*_ should be a promising choice to capture the important local facial regions for FER (He et al., 2021). Thus, we can choose the positions of the maximum values with regard to the *M* different attention heads in **a**_*total*_. We then choose the indexes of the maximum values *A*_1_, *A*_2_, ⋯ , *A*_*M*_ w.r.t the *M* different attention heads in **a**_*total*_. These indexes are exploited as positions for RS-Unit to select the corresponding tokens in **Z**_*L*_. At last, we combine the classification token with the selected tokens along as the final representation:


(5) $$\begin{array}{l} Z_{select}=\text{Concat}\left[Z_{L}^{0},Z_{L}^{A_{1}},Z_{L}^{A_{2}},\cdots \;,Z_{L}^{A_{M}}\right]. \end{array}$$

By utilizing the entire input sequence with tokens tightly related to discriminative facial regions and combine the classification token as input to the classification layer, our proposed TFE is capable of utilizing the global facial information but also the local facial regions that contain critical subtle facial deformations induced by facial expressions. Thus, our proposed TFE is expected to perceive the discriminative facial regions for robust FER under occlusions.

### 3.3. Image Reconstruction

Since the facial expression is a subtle deformation of faces that can be inferred from multiple facial regions, it is beneficial to explicitly infer the occluded facial parts from the unoccluded regions. In the image inpainting process, the model is tasked to precisely perceive the fine-grained facial action units to infer their co-occurrence (Li et al., 2018a).

**Figure 3:**
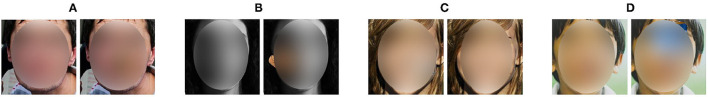
Examples of the synthesized occluded images. The occluders are various in shape, color, and facial positions. **(A)** Anger, **(B)** neutral, **(C)** happy, and **(D)** sad.

Inspired by this, we propose to reconstruct the facial image with an auxiliary decoder. To this end, we synthesize the occluded face images by manually collecting abundant masks for generating the occluders. We show some randomly selected occluded images in Figure 3. With the occluded faces **I**_*occ*_ and the corresponding original images **I**_*ori*_, we are capable of reconstructing the images as follows,


(6) $${ℒ}_{rec}=\|I_{ori}-Dec(Enc(I_{occ})){\|}_{1},$$

where *Enc* means the convolutional feature extraction operation shown in Figure 2, *Dec* denotes the image decoding process.

### 3.4. Overall Objective

Transformer-based FER method is trained in an end-to-end fashion by minimizing the integration of the FER loss and the image reconstruction loss in Equation (6). We integrate the two goals and obtain the full objective function:


(7) $${ℒ}_{total}={ℒ}_{cls}+\lambda {ℒ}_{rec},$$

where hyper-parameter λ controls the importance of the image reconstruction term.

## 4. Experiment

### 4.1. Implementation Details

We adopted ResNet-18 (He et al., 2016) as the backbone network for TFE due to its elegant structure and excellent performance in image classification. We used the output of the third stage as the convolutional feature maps: **X** ∈ ℝ^14×14×1024^. Thus, the token size is *N* = 14 × 14. We set *L* = 4, *D* = 768, and *M* = 12. We initialized the backbone of TFE with the pre-trained model based on ImageNet dataset. We mixed all the facial expression datasets with their modifications with artificial facial occlusions with the ratio of 1:1. TFE was optimized *via* a batch-based stochastic gradient descent manner. We actually set the batch size as 128 and the base learning rate as 0.001. The weight decay was set as 0.0005 and the momentum was set as 0.9. The optimal setting for the loss weight between the FER and image reconstruction term was set as 1:1 by grid search.

#### 4.1.1. Datasets

We evaluated the methods on two facial expression datasets [RAF-DB (Li et al., 2017) and AffectNet (Mollahosseini et al., 2017)]. We additionally evaluate our proposed TFE on FED-RO dataset (Li et al., 2018a). **RAF-DB** consists of about 30,000 facial images annotated with compound or basic expressions by 40 trained human. We merely used the images with seven basic expressions. We obtained totally 12,271 images for training data and 3,068 images for evaluation. **AffectNet** is currently the largest dataset with annotated facial expressions. AffectNet consists of approximately 400,000 images manually annotated. We merely utilized the images with six basic and neutral expressions, We obtained about 280,000 images for training and 3,500 images for evaluation. **FED-RO** (Li et al., 2018a) is a facial expression database with real-world occlusions. Each face has real occlusions in uncontrolled environment. There are totally 400 images in FED-RO dataset annotated with seven expressions. We train the proposed TFE on the joint training data of AffectNet and RAF dataset, following the protocol suggested in Li et al. (2018a).

Following (Li et al., 2018a), we manually collected approximately 4 k images as masks for generating the occluders. These occluders were discovered and saved from search engine *via* more than 50 keywords, such as hair, hat, book, beer, apple, cabinet, computer, orange, etc. The height *H* and width *W* of the occluders *S* satisfy *H* ∈ [96, 128] and *W* ∈ [96, 128]. Figure 3 shows some occluded faces. It is evident that the artificial occluded facial images are diverse in occlusion patterns.

#### 4.1.2. Evaluation Metric

We report FER performance on both the occluded and non-occluded images of all the datasets. We used the overall and the overall and average accuracy on seven facial expression categories (i.e., six prototypical plus neutral categories) as a performance metric. Besides, we also report some confusion matrixes on RAF-DB dataset to show the discrepancies between the expressions.

### 4.2. FER Experimental Results

We compare the proposed TFE with the state-of-the-art FER methods, including DLP-CNN (Li et al., 2017), gACNN (Li et al., 2018a), FAB-Net (Wiles et al., 2018), TAE (Li et al., 2020), OADN (Ding et al., 2020), and SCN (Wang et al., 2020a). The comparison results are shown in Tables 1–**3**.

**Table 1:** Test set accuracy on RAF-DB dataset.

**Method**	**Neutral**	**Anger**	**Disgust**	**Fear**	**Happy**	**Sad**	**Surprise**	**ACC (Overall/Ave)**
AlexNet (Li et al., 2017)	60.15	58.64	21.87	39.19	86.16	60.88	62.31	−/55.60
VGG16 (Li et al., 2017)	59.88	68.52	27.50	35.13	85.32	64.85	66.32	80.96/58.22
DLP-CNN (Li et al., 2017)	80.29	71.60	52.15	62.16	92.83	80.13	81.16	80.89/74.20
gACNN (Li et al., 2018a)	84.30	78.42	53.11	55.39	93.17	82.88	86.27	85.07/76.22
TAE (Li et al., 2020)	62.80	58.01	45.03	58.12	76.03	45.85	64.44	81.68/58.61
TFE (Ours)	86.76	79.01	64.38	66.22	95.61	87.03	90.27	88.49/81.33

Table 1 shows the FER results of our method and previous studies on RAF-DB dataset. Our TFE achieves 81.33% in the average accuracy on seven facial expression categories. Compared with DLP-CNN (Li et al., 2017), TFE obtains 7.13% improvements in the average accuracy. Compared with the strongest competing method in the same setting gACNN (Li et al., 2018a), TFE surpasses it by 5.61%. The benefits of TFE over other methods can be explained in two-fold. First, TFE explicitly utilizes transformer layers in the network structure. The self-attention in the transformers has been shown to effectively learn local to global interactions and relations between distant facial parts. Besides, the RS-Unit on top of the transformer layers in our proposed TFE helps perceive the critical facial regions. Thus, TFE is capable of spotting the local subtle facial deformations induced by facial expressions. Second, TFE explicitly reconstructs the unoccluded facial images with an auxiliary decoder, which facilitates the backbone CNN in TFE to learn to infer the occluded facial parts *via* the important facial regions.

Table 2 shows the comparisons of our TFE and other state-of-the-art FER methods on AffectNet dataset. TFE achieves 63.33% in the average accuracy on seven facial expression categories. Compared with RAN-ResNet-18 (Wang et al., 2020b) that use multiple crops of facial images as input and learns adaptive weights for each input image, TFE obtains 10.43% improvements in the average accuracy. Compared with the self-supervised methods FAB-Net (Wiles et al., 2018) and TAE (Li et al., 2020), TFE shows its success in almost each facial expression category. Among the state-of-the-art FER methods, gACNN (Li et al., 2018a) and OADN (Ding et al., 2020) both exploit the 24 facial landmarks for facial region decomposition and learn the path-specific representation to better capture the local details of the input facial image. However, their FER performance still lags behind our proposed TFE, as illustrated in Table 2. This is because the transformer layers in TFE naturally encode the patch-specific face representation by tokenizing the input convolutional feature maps. TFE does not rely on facial landmarks to extract the local representations and avoids the negative influence induced by the misalignments of the facial landmarks. We additionally show the FER performance comparison on FED-RO dataset in Table 3. FED-RO dataset is the first facial expression dataset with real occlusions. TFE achieves 70.60% in the average accuracy and outperforms other compared methods with no exception. In summary, the experimental results in Tables 1–3 verify the superiority of the proposed TFE for robust facial expression recognition.

**Table 2:** Validation set accuracy on AffectNet dataset.

**Method**	**Neutral**	**Anger**	**Disgust**	**Fear**	**Happy**	**Sad**	**Surprise**	**ACC (Overall/Ave)**
AlexNet (Mollahosseini et al., 2017)^*^	−	−	−	−	−	−	−	47.00/47.00
RAN-ResNet18 (Wang et al., 2020b)^*^	−	−	−	−	−	−	−	52.90/52.90
VGG16 (Simonyan and Zisserman, 2014)	**89.61**	53.42	20.61	32.03	90.03	35.01	37.22	51.13/51.13
FAB-Net (Wiles et al., 2018)	38.64	30.62	**48.42**	32.14	82.25	35.61	51.42	45.59/45.59
TAE (Li et al., 2020)	44.42	38.63	46.84	40.39	78.01	40.81	**54.41**	49.07/49.07
gACNN (Li et al., 2018a)	73.42	66.18	32.59	46.22	93.81	55.82	43.43	58.78/58.78
OADN (Ding et al., 2020)	−	−	−	−	−	−	−	61.90/61.90
SCN (Wang et al., 2020a)	−	−	−	−	−	−	−	60.23/60.23
TFE (Ours)	76.03	**68.09**	46.83	**47.03**	**94.12**	**57.32**	53.90	**63.33/63.33**

*The bold values denotes the best results*.

* *Means the values are reported in the original papers*.

**Table 3:** Test set accuracy on FED-RO dataset.

**Method**	**ResNet18**	**RAN**	**DLP-CNN**	**gACNN**	**OADN**	**TFE**
ACC (AVE)	64.25	67.98	60.31	66.50	68.11	**70.60**

*The bold values denotes the best results*.

#### 4.2.1. Ablation Study

Both the transformer layers and auxiliary decoder help TFE obtain improvements on FER. We performed a quantitative study of these two parts in order to better understand the benefits of TFE.

We show the FER performance of TFE without auxiliary image reconstruction decoder and without the transformer layers (as well as RS-Unit) [TFE (*w/o D, w/o T*)], and TFE with the auxiliary image reconstruction decoder but without transformer layers and RS-Unit [TFE (*w/ D, w/o T*)] in Table 4. It is clear that TFE (*w/o D, w/o T*) shows decreased FER performance on both the original and synthesized occluded face images. With the auxiliary image reconstruction decoder, TFE (*w/ D, w/o T*) illustrates improved FER performance in many facial expression categories. The comparisons between TFE (*w/o T, w/o D*) and TFE (*w/ T, w/o D*) demonstrate the effectiveness of the auxiliary image reconstruction decoder. With the transformer layers and the auxiliary image decoder, TFE obtains the best FER performance. As illustrated in Table 4, TFE shows its benefits in *Neutral, Fear, Surprise* and obtains comparable accuracy in *Disgust, Happy, Sad*.

**Table 4:** Ablation study on RAF-DB dataset.

**Method**	**Neutral**	**Anger**	**Disgust**	**Fear**	**Happy**	**Sad**	**Surprise**	**ACC (Overall/Ave)**
**Original test set of RAF-DB dataset**								
TFE (*w/o D, w/o T*)	83.97	79.01	60.63	60.81	94.51	85.56	86.32	85.91 /79.69
TFE (*w/ D, w/o T*)	85.15	**83.33**	**65.63**	64.86	**95.78**	**87.03**	84.80	86.20/80.94
**TFE**	**86.76**	79.01	64.38	**66.22**	95.61	**87.03**	**90.27**	**88.64/81.33**
**Synthesized occluded test set of RAF-DB dataset**								
TFE (*w/o D, w/o T*)	79.41	**76.54**	53.12	54.05	91.90	81.80	80.85	83.68/73.95
TFE (*w/ D, w/o T*)	81.47	75.93	55.62	59.46	93.42	**84.73**	80.55	84.00/75.88
**TFE**	**83.53**	72.84	**60.00**	**67.57**	**93.50**	82.85	**85.41**	**85.12/77.96**

*The bold values denotes the best results*.

**Figure 4:**
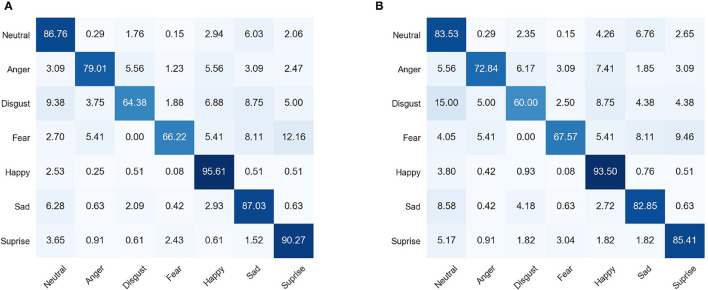
Confusion matrixes of TFE. **(A)** Denotes the confusion matrix for the original test set of RAF-DB. **(B)** Is the confusion matrix for the synthesized occluded test set of RAF-DB. It is clear that TFE shows decreased performance on most of the facial expression categories with the manually occluders in the facial images.

We additionally show the confusion matrixes of our proposed TFE on both the original and synthesized occluded test set of RAF-DB dataset in Figure 4. It is clear that TFE shows degraded performance on most of the facial expression categories when the facial images are occluded in Figure 4B. Besides, TFE shows the lowest FER accuracy on *Disgust* category and highest accuracy on *Happpy* category. Easily confused expression categories are *disgust* and *sad, fear* and *surprise*, and *fear* and *sad*. Our above observations are consistent with the conclusions in Li et al. (2018a).

**Figure 5:**
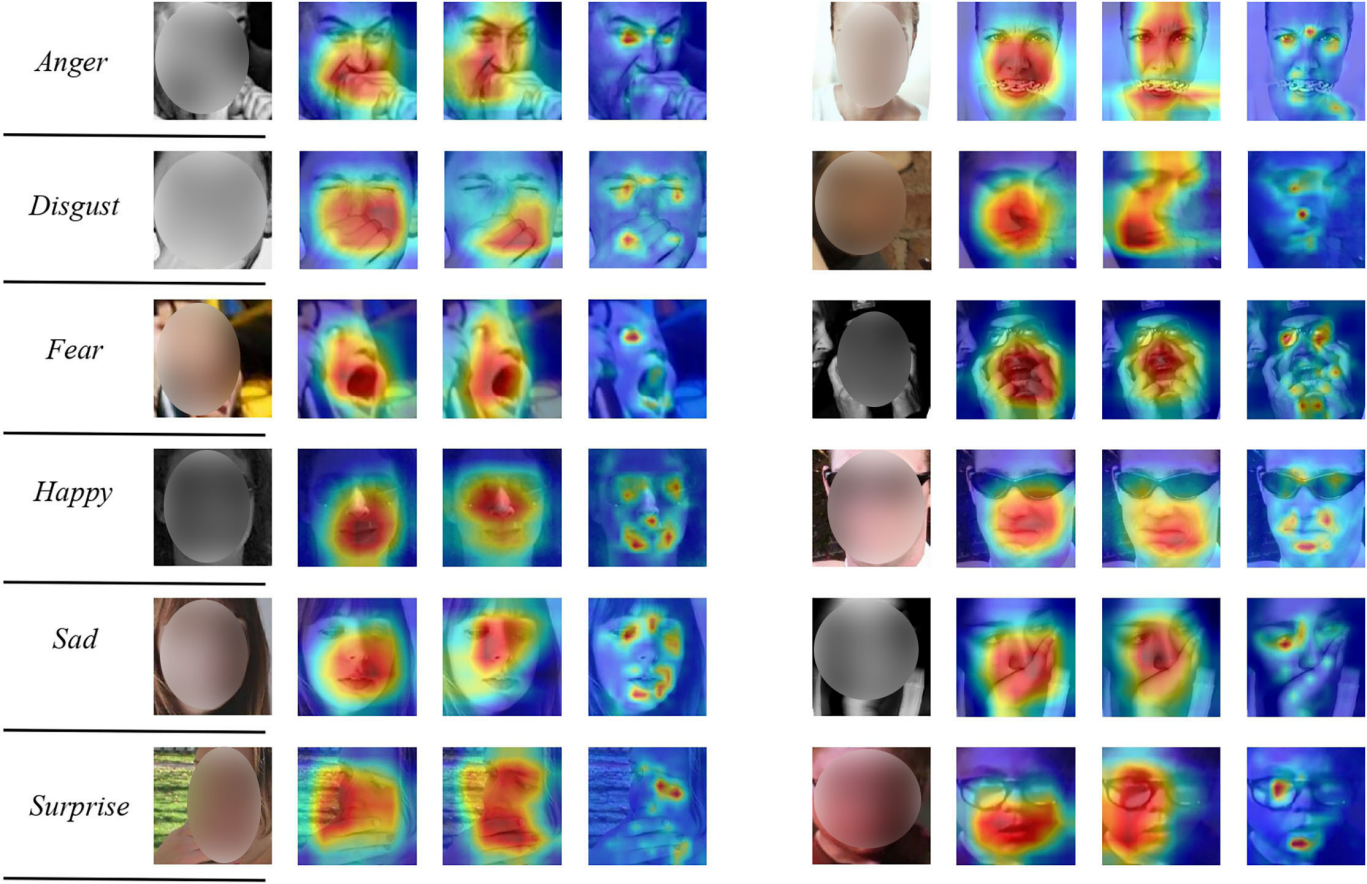
Attention maps of several facial images with occlusions. For each input face image, the first, second, and third column, respectively, show the attention map of TFE (*w/o D, w/o T*), TFE (*w/ D, w/o T*), and TFE. Our proposed TFE is capable of perceiving the important facial regions for robust FER. A deep red denotes low attention. A deep red means high attention. Better viewed in color and zoom in.

We show the attention maps of the TFE and its variants in Figure 5. For each input face, the first, second, and third column, respectively, show the attention map of TFE (*w/o D, w/o T*), TFE (*w/ D, w/o T*), and our proposed TFE. It is evident that TFE is capable of shifting attention from the occluded facial patches to other unobstructed regions. As a comparison, TFE (*w/o T, w/o D*) and TFE (*w/ D, w/o T*) are not capable of precisely focusing on the important and unobstructed facial parts. Taking facial images labeled with *Happy* in the fourth row for example, TFE perceives the eyes and the corner or the mouth precisely, irrespective of the facial occlusions. The visualization results show the benefits of the proposed RS-Unit and the auxiliary decoder for robust FER under occlusions.

## 5. Conclusions

In this study, we propose a transformer-based FER method (TFE) that is capable of adaptatively focusing on the most important and unoccluded facial regions. Considering that facial expression is represented by several specific facial parts, we propose a RS-Unit to automatically perceive the critical facial parts so as to explicitly perceive the important facial regions for robust FER. To better perceive the fine-grained facial deformations and infer the co-occurrence of different facial action units, TFE consists of an auxiliary decoder to reconstruct the facial image. Quantitative and qualitative experiments have verified the feasibility of our proposed TFE. TFE also outperforms other state-of-the-art FER approaches. Ablation and visualization analyses show TFE is capable of shifting attention from the occluded facial regions to other important ones. Currently, TFE exploits the fixed patch size as the input to the transformer layer while larger facial patch size might be a better choice for the heavily occluded facial images. We will explore this in the future work. Besides, we will also explore how to reduce the computation overhead and make TFE suit for mobile deployment.

## Data Availability Statement

The original contributions presented in the study are included in the article/supplementary material, further inquiries can be directed to the corresponding author/s.

## Author Contributions

JG and YZ cooperatively led the method design and experiment implementation. JG wrote the sections of the manuscript. YZ provided result review, theoretical guidance, and paper revision. Both authors have read and approved the final manuscript.

## Funding

This publication of this paper was supported by the Henan key R & D and promotion projects (Grant: 212102310551) and the Key Scientific Research Project Plan of Henan Province colleges and universities (19A520008, 20A413002).

## Conflict of Interest

The authors declare that the research was conducted in the absence of any commercial or financial relationships that could be construed as a potential conflict of interest.

## Publisher's Note

All claims expressed in this article are solely those of the authors and do not necessarily represent those of their affiliated organizations, or those of the publisher, the editors and the reviewers. Any product that may be evaluated in this article, or claim that may be made by its manufacturer, is not guaranteed or endorsed by the publisher.
